# Urban Legends and Paranormal Beliefs: The Role of Reality Testing and Schizotypy

**DOI:** 10.3389/fpsyg.2017.00942

**Published:** 2017-06-08

**Authors:** Neil Dagnall, Andrew Denovan, Kenneth Drinkwater, Andrew Parker, Peter J. Clough

**Affiliations:** Department of Psychology, Manchester Metropolitan UniversityManchester, United Kingdom

**Keywords:** urban legends, paranormal belief, reality testing, schizotypy

## Abstract

Recent research suggests that unconventional beliefs are locatable within a generic anomalous belief category. This notion derives from the observation that apparently dissimilar beliefs share fundamental, core characteristics (i.e., contradiction of orthodox scientific understanding of the universe and defiance of conventional understanding of reality). The present paper assessed the supposition that anomalous beliefs were conceptually similar and explicable via common psychological processes by comparing relationships between discrete beliefs [endorsement of urban legends (ULs) and belief in the paranormal] and cognitive-perceptual personality measures [proneness to reality testing (RT) and schizotypy]. A sample of 222 volunteers, recruited via convenience sampling, took part in the study. Participants completed a series of self-report measures (Urban Legends Questionnaire, Reality Testing subscale of the Inventory of Personality Organization, Revised Paranormal Belief Scale and the Schizotypal Personality Questionnaire Brief). Preliminary analysis revealed positive correlations between measures. Within schizotypy, the cognitive-perceptual factor was most strongly associated with anomalistic beliefs; disorganized and interpersonal produced only weak and negligible correlations respectively. Further investigation indicated complex relationships between RT, the cognitive-perceptual factor of schizotypy and anomalistic beliefs. Specifically, proneness to RT deficits explained a greater amount of variance in ULs, whilst schizotypy accounted for more variance in belief in the paranormal. Consideration of partial correlations supported these conclusions. The relationship between RT and ULs remained significant after controlling for the cognitive-perceptual factor. Contrastingly, the association between the cognitive-perceptual factor and ULs controlling for RT was non-significant. In the case of belief in the paranormal, controlling for proneness to RT reduced correlation size, but relationships remained significant. This study demonstrated that anomalistic beliefs vary in nature and composition. Findings indicated that generalized views of anomalistic beliefs provide only limited insight into the complex nature of belief.

## Introduction

Contemporary/modern urban legends (ULs) are widely circulated, unauthenticated narrative accounts of rare or bizarre events that convey warnings or cautionary advisements (Guerin, 2003, 2004; DiFonzo and Bordia, 2007). Accordingly, ULs typically contain sensational/dramatic content intended to prompt strong emotional reactions within recipients (i.e., horror, shock, revulsion, and humor) (Heath et al., 2001). The study of ULs is important academically because they represent enduring social narratives, which reach wide audiences and potentially influence significant numbers of people. Indeed, many receivers incorrectly believe that ULs contain factual material (Brunvald, 1981; Fox Tree and Weldon, 2007). A commonly cited example is the apocryphal tale that mature alligators, flushed down the toilet as hatchlings, now inhabit sewers and pose a threat to urban dwellers (Mullen, 1972).

At a social level, ULs become resistant to abjuration and persist because of regular retelling. Declarations within ULs, stating that there is a risk attached to recipients failing to pass their content on, serve also to facilitate cultural propagation (Dagnall et al., 2010). Correspondingly, over time, ULs become part of social record, occupy societal awareness and form part of public consciousness (Larrington, 2015). Hence, ULs are readily available and frequently encountered. Furthermore, despite refutation and counter-evidence, ULs periodically re-surface. This is particularly true in contemporary society, where email and social media ensure that ULs circulate indefinitely (Conn, 2008). Within this self-perpetuating process, narratives evolve and adapt to accommodate contradictory evidence (Inglis, 2007).

Urban legends possess other important features. Particularly, themes remain somewhat constant, whilst precise details vary and adapt (e.g., place names and/or topographical information). A classic example of this is the existence of campus legends (Brunvand, 2012). These are generic stories, which adjust to the characteristics of particular educational institutions (Tucker, 2005). Similarly, content alters over time (e.g., ancient battlefield stories reference recent or present conflicts) and often embraces social and technological advancements (computer viruses, global warning, etc.). These modifications in surface structure ensure that ULs remain relevant, coherent and significant (Fox Tree and Weldon, 2007).

To date, relatively few psychological studies have focused on predictors and correlates of endorsement of ULs (Dagnall et al., 2010; Drinkwater et al., 2012). This is surprising because of the social importance of ULs and the fact they share key psychological features with other more widely researched atypical beliefs (i.e., paranormal and conspiracy-related ideation). This view is congruent with recent work on ‘anomalous belief,’ which focuses on the reasons why people accept and endorse unusual/atypical beliefs, experiences and behaviors as real and authentic. This perspective originates from the assumption that anomalous beliefs are those, which contradict orthodox scientific understanding of the universe and defy conventional understanding of reality (French and Stone, 2013; Brotherton and French, 2014).

This classification draws on Irwin’s (2009, pp 16–17) delineation of paranormal belief as, “a proposition that has not been empirically attested to the satisfaction of the scientific establishment but is generated within the non-scientific community and extensively endorsed by people who might normally be expected by their society to be capable of rational thought and reality testing.” In this context, reality testing (RT) refers to the ability to assess the validity of beliefs and suppositions via reference to external sources of information. Thus, RT relies (in part) on the ability to monitor and distinguish accurately between external and internal sources of information. Indeed, investigations report that believers demonstrate a preference for subjective-intuitive thinking style (vs. rational-analytical) and possess a tendency to report proneness to RT deficits (Dagnall et al., 2010). For example, Aarnio and Lindeman (2005) reported that intuitive thinking was positively associated with belief in the paranormal.

Previous research uses the notion that subjective/personal experiences guide and structure interpretation of information to explain belief in the paranormal (Epstein et al., 1996; Wolfradt et al., 1999). From the believers’ perspective, intuitions and beliefs are self-evidently valid, and accordingly exempted from critical scrutiny (Epstein et al., 1996; Stanovich and West, 2000). Concomitantly, participants demonstrating higher levels of analytic reasoning are less likely to validate supernatural beliefs (Pennycook et al., 2012).

Cognitive-experiential self-theory (CEST, e.g., Epstein, 1990, 1994) embodies these ideas and delineates clearly between experiential and rational processing. Experiential thinking style is fast, automatic, holistic and characterized by proneness to generalization/association. Experiential thinking draws heavily on previous experience, prefers emotional appeal, and is highly resistant to change. Contrastingly, rational thinking is slow, intentional, effortful and logical; conscious cognitive appraisal mediates decision-making (Epstein, 1990, 1993). CEST provides a useful framework for explaining belief in the paranormal (Lasikiewicz, 2016).

In support of a link between intuitive-experiential thinking and general endorsement of anomalous beliefs, Dagnall et al. (2010) found that attributes of ULs (truthfulness, retelling, likelihood, importance, scariness, strangeness, and heard by others) correlated positively with RT scores and belief in the paranormal. The best predictor of perceived veracity was proneness to RT deficits. Overall, findings were consistent with Irwin’s (2003, 2004) supposition that validation of anomalous beliefs was associated with intuitive-experiential thinking style and the failure to subject data (evidence, experiences, thoughts, etc.) to critical analytical-rational processing. Hence, belief in ULs may arise from an overreliance on subjective (vs. objective) evidence.

Considering ULs in the context of Irwin’s definition of paranormal belief, endorsement of the two phenomena share important intuitively ‘apparent’ similarities. Firstly, ULs originate outside the scientific community (their precise origin is generally unknown) (Harding, 2016). Secondly, narratives contain dubious, unsubstantiated evidence and their legitimacy is unattested (Brunvand, 2012). Thirdly, by virtue of widespread dissemination, people who typically engage in cogent thought and are capable of RT endorse ULs (Dagnall et al., 2010).

Whilst correspondences suggest significant conceptual intersection between ULs and paranormal beliefs, research reveals only a weak to moderate association. For example, Dagnall et al. (2010) observed a small positive correlation (*r* = 0.28), indicating that the constructs shared only 8% variance. Drinkwater et al. (2012), using the Australian-Sheep Goat Scale (ASGS; Thalbourne and Delin, 1993) and the Revised Paranormal Belief Scale (R-PBS; Lange et al., 2000), reported common variance ranging between 14 and 30%. This proposes that other cognitive-perceptual factors (alongside preferential thinking style) also influence validation of ULs.

The current paper extended previous work by including a measure of schizotypy alongside proneness to RT deficits. The addition of schizotypy was useful because it is a factor generally associated with inclination to odd and unusual beliefs (Barlow et al., 2009; Darwin et al., 2011). Specifically, schizotypy is a multidimensional psychological construct comprising cognitive, perceptual and affective dimensions that represent vulnerability to schizophrenia-spectrum pathology (Mason and Claridge, 2015). The model of schizotypy developed within different psychological-related sub-disciplines (e.g., individual differences and medical traditions). Hence, various definitions exist: the personality, quasi-dimensional, and fully dimensional models (Barrantes-Vidal et al., 2015). The quasi-dimensional or disease model (Rado, 1953; Meehl, 1962) depicts schizotypy as a milder form of schizophrenia (Goulding, 2004). Contrastingly, the personality framework developed by Eysenck (1960) views psychoticism as the upper end of the normality-psychosis continuum. These approaches are reconciled within the fully dimensional model (Claridge, 1997), which designates schizotypy as continuously distributed trait (a form of healthy variation and predisposition to psychosis) (Goulding, 2004).

Personality-based models are pertinent to the present article because they advocate relationships between level of schizotypy, cognitive-perceptual processing and openness to anomalous beliefs (Dagnall et al., 2010). This view is consistent with empirical evidence. For example, Simmonds-Moore (2010) noted that individuals scoring higher on positive schizotypy possessed stronger belief in anomalous phenomena. Several studies report similar associations between schizotypy and belief in the paranormal (Genovese, 2005; Hergovich and Arendasy, 2007; Hergovich et al., 2008). This association, however, varies as a function of belief type (Irwin and Green, 1998-1999). Cognitive-perceptual scores correlate with New Age Philosophy (psychic ability and spiritualism) and interpersonal with belief in extraordinary life forms and witchcraft. Contrastingly, disorganization influences evaluation of paranormal experiences and relates less to belief in the paranormal (Schofield and Claridge, 2007; Irwin, 2009). The influence of schizotypy extends also to conspiratorial ideation (Barron et al., 2014; Dagnall et al., 2015). In support of this, Dagnall et al. (2015) found positive schizotypy correlated with conspiracist beliefs.

The present paper assessed the notion that unconventional beliefs were locatable within a generic anomalous belief category. The veracity of this supposition presupposed that endorsement of ULs and belief in the paranormal would highly positively correlate, and that belief types would relate similarly to cognitive-perceptual factors (proneness to reality deficits and schizotypy). Whilst parsimonious, the proposed anomalous classification is somewhat reductionist and inconsistent with previous evidence. In this context, evaluation of the model provided a means by which to extend understanding of unconventional beliefs. This was conceptually and methodologically important because researchers are increasingly using the anomalous classification to group discrete unconventional beliefs. For example, Brotherton and French (2014) deduced commonality between endorsement of conspiracy theories and belief in the paranormal. Specifically, they claimed that conspiracy theories, like other anomalous beliefs, were associated with reasoning and heuristical bias. This conclusion, possesses intuitive appeal, but overstates the case. Considered in the setting of the present study, it was evident that whilst surface similarities (i.e., defiance of conventional understanding of reality) suggested overlap between UL endorsement and belief in the paranormal, preceding work indicated only modest shared variance (Dagnall et al., 2010; Drinkwater et al., 2012).

Indeed, careful consideration of the properties of the constructs showed significant divergence and suggested differential interactions with cognitive-perceptual factors. Explicitly, because validation of ULs derives (largely) from acceptance of unreliable, dubious information as accurate and authentic, a stronger relationship was anticipated between endorsement of ULs and the reality monitoring measure (vs. schizotypy). Contrastingly, since belief in the paranormal is a broad, diffuse construct, embracing multifarious phenomenon (traditional religious belief, psi, witchcraft, superstition, spiritualism, extraordinary life forms, and precognition) the researchers anticipated stronger associations between belief in the paranormal and schizotypy (vs. RT). This prediction was consistent with the observation that schizotypy influences perception of causality and connectedness. Indeed, positive schizotypy (as measured by the cognitive-perceptual factor of the SPQ-B) is associated with illusory causation, particularly magical ideation (Rominger et al., 2011). These properties were likely to be particularly attendant with belief in the paranormal because validation of some facets of paranormal depends on acceptance of odd and unusual relationships. From this perspective, the inclusion of schizotypy alongside RT was justified and appropriate.

## Materials and Methods

### Design

A correlational design was used in which (proneness to RT deficits and schizotypy) were used as predictors of anomalous beliefs (endorsement of UL truthfulness and belief in the paranormal). The design extended beyond consideration of simple linear relationships to include assessment of structural relationships within measurement models.

### Respondents

In total 222 respondents participated in this study, 62 (28%) males and 160 (72%) females. Mean overall age was 30.77 years (*SD* = 11.74), with a range of 16–63 years; male *M* = 33.00, range 17–61 years *SD* = 13.15, female *M* = 29.9, range 16–63 years *SD* = 11.06. Respondents included undergraduates and employees from the Manchester Metropolitan University (MMU) and members of the wider community. Recruitment was via emails and posters to university staff/students and local stakeholders (businesses, leisure, and vocational/sports classes). Overall, 60% of respondents were students and 40% non-students. Exclusion criteria indicated that respondents should participate only if they were at least 18 years of age and had not previously completed a previous study on ULs.

### Measures

Respondents completed booklets containing the following self-report measures: Urban Legends Questionnaire (ULQ) (Dagnall et al., 2010); RT subscale of the Inventory of Personality Organization (IPO-RT; Lenzenweger et al., 2001), R-PBS (Tobacyk and Milford, 1983; Tobacyk, 1988; Lange et al., 2000; Tobacyk, 2004) and the Schizotypal Personality Questionnaire Brief (SPQ-B, Raine and Benishay, 1995). To control for order effects counterbalancing rotated scale order across respondents.

### Urban Legends Questionnaire (ULQ)

The ULQ contains seven ULs (Kidney, Cactus, Cookie, Airbag, Airplane, Ricin, and Sandstorm) based on Fox Tree and Weldon (2007). Detail within ULs derived from the Snopes online database^1^. Each item within the ULQ possesses the same underlying structure. A narrative outlined the UL in detail (in the form of a story, email, warning, article, etc.) (cf. Dagnall et al., 2010). Presentation within the ULQ is similar to that observed within real world situations (e.g., email, internet, and traditional media sources). A series of questions followed each narrative. The opening question, asked whether respondents had heard the story before and, if so, how many times and where. The second, assessed whether respondents believed other people in the United Kingdom had heard the story (heard by others), 1 (*almost no one*) to 7 (*almost everyone*). A succeeding item enquired whether the respondent would retell the story in the future (retelling), 1 (*not at all likely*) to 7 (*extremely likely*). The third question evaluated whether respondents believed the story was true (truthfulness), 1 (*definitely not true*) to 7 (*definitely true*). Item four measured the extent to which respondents thought something similar to that depicted in the narrative could happen to someone they knew (likelihood), 1 (*not at all likely*) to 7 (*extremely likely*). Question 5 gauged whether respondents believed it was important to pass on the story (importance), 1 (*not at all important*) to 7 (*extremely important*). The penultimate item ascertained whether the respondent considered the story frightening (scariness), 1 (*not at all*) to 7 (*extremely*). The final question asked whether respondents believed the event was out of the ordinary (strangeness), 1 (ordinary/*not at all unusual*) to 7 (*extremely unusual*). Use of the full question set ensured that respondents engaged fully with individual ULs. For the purpose of this study, only overall endorsement of truthfulness was of interest. Thus, UL scores ranged from 7 to 49, with higher scores indicating greater endorsement.

### Reality Testing

The RT subscale of the IPO-RT (Lenzenweger et al., 2001) is a 20 item, unidimensional, self-report measure, which assesses proneness to RT deficits. Particularly, “the capacity to differentiate self from non-self, intrapsychic from external stimuli, and to maintain empathy with ordinary social criteria of reality” (Kernberg, 1996, p. 120). Responses indicate agreement to statements on a five-point likert scale (1 = never true, to 5 = always true). Total scores range from 20 to 100, with low scores indicating high RT ability. The IPO-RT focuses on upon information processing style rather than psychotic symptomology (Langdon and Coltheart, 2000; Irwin, 2004). The subscale possesses established psychometric integrity. Lenzenweger et al. (2001) report the scale is internally consistent and temporally stable with non-clinical populations. Indeed, the IPO-RT has demonstrated decent retest reliability (*r* = 0.73) and good construct validity (Lenzenweger et al., 2001).

### The Revised Paranormal Belief Scale (R-PBS) (Tobacyk and Milford, 1983; Tobacyk, 1988, 2004; Lange et al., 2000)

The RPBS is the most prevalently used self-report measure of paranormal belief (Irwin, 2004). It is an amended form of the Paranormal Belief Scale developed by Tobacyk and Milford (1983) and contains 26-items assessing seven facets of paranormal belief: traditional religious belief, psi, witchcraft, superstition, spiritualism, extraordinary life forms, and precognition. Items on the R-PBS are presented as statements (e.g., “there is a devil” and “witches do exist”) and participants respond on a likert scale ranging from 1 (strongly disagree) to 7 (strongly agree), with higher scores reflecting greater paranormal belief. Summated items produce subscale and overall scores. Alternatively, purification of the scale to correct for differential item functioning (arising from age and gender bias), identified a two factor solution (Lange et al., 2000). This comprises factors measuring New Age Philosophy (NAP) and Traditional Paranormal Belief (TPB). NAP contains 11 items measuring belief in psi, reincarnation, altered states, and astrology, whilst the TPB assesses belief in concepts, such as the devil and witchcraft (Irwin, 2004). These dimensions reflect belief functions (individual vs. social) (Lange et al., 2000). NAP imparts control over external events (Irwin, 1992), whilst TPB regulates social/cultural factors (Goode, 2000). Recoding in line with Rasch scaling procedure (Andrich, 1988) produces scores ranging from 6.85 to 47.72 on NAP and 11.16 to 43.24 on TPB. Despite debate about the nature and number of belief dimensions contained within the R-PBS (Lawrence, 1995a,b; Tobacyk, 1995a,b; Lawrence et al., 1997; Tobacyk and Thomas, 1997), the measure is conceptually and psychometrically satisfactory (Tobacyk, 2004). Particularly, the R-PBS possesses adequate validity (Tobacyk, 1995a,b, 2004) and good test–retest reliability (Tobacyk, 2004).

### The Schizotypal Personality Questionnaire (SPQ-B)

The SPQ-B (Raine and Benishay, 1995) is an easy-to-administer 22-item instrument for assessing level of schizotypy. It is a briefer version of the 74-item SPQ and comprises items from three subscales: cognitive-perceptual, eight items; interpersonal, eight items; and disorganized, six items. The SPQ-B correlates highly with the full version and features prominently within published research (Bailey and Swallow, 2004). The present study employed the SPQ-B because of its brevity. The SPQ-B possesses psychometric integrity, good internal consistency reliability, test–retest reliability, and criterion validity (Raine and Benishay, 1995). Raine and Benishay (1995) found the internal reliabilities of the subscales ranged from 0.72 to 0.80, with a mean of 0.76. Similarly, Axelrod et al. (2001) observed reliabilities ranging from (0.74 to 0.76). The SPQ-B contains statements responded to with “yes” or “no” answers. Yes-responses are totaled to produce an overall score ranging from 0 to 22; higher scores specify higher levels of self-reported schizotypy.

### Procedure

Potential respondents read the study background information before deciding whether to participate. This stated that the research was concerned with beliefs in unusual phenomena and cognitive-perceptual personality factors. Respondents who agreed indicated informed consent and received the materials booklet. Instructions told participants to take their time and answer questions openly and honestly. The booklet contained five subdivisions: demographic information (completed first), ULs, belief in the paranormal, RT and the SPQ-B. Scale order rotated across respondents.

## Results

### Justification and General Analytical Strategy

Data analysis progressed systematically through a series of stages. Initially, consideration of zero-order inter-measure correlations specified relationships between variables. Next, confirmatory factor analysis and composite reliability assessed the adequacy of measurement models. Finally, structural relationships among measurement models were tested. Specifically, the degree to which schizotypy and RT predicted belief in the paranormal and endorsement of ULs.

Separate analysis of TPB and NAP was undertaken because, whilst typically related, researchers contend that these dimensions satisfy distinct functions (Lange et al., 2000). This approach was consistent with prior research, which observed dissimilar relationships between dimensions of belief in the paranormal and cognitive-perceptual factors (Dagnall et al., 2016b). Analysis tested four models. Model 1 considered schizotypy in relation to TPB and ULs. Model 2 modified Model 1, replacing TPB with NAP. Model 3 examined RT as a predictor of TPB and ULs, and Model 4 evaluated RT as a predictor of NAP and ULs.

Analysis compared data fit and the predictive power of each model via consideration of several fit indices. These included chi-square (χ^2^), the Comparative Fit Index (CFI), the Standardized Root-Mean-Square Residual (SRMR) and the Root-Mean-Square Error of Approximation (RMSEA). Chi-square is a widely cited, traditional measure of overall model fit (Hu and Bentler, 1999). An insignificant result at a 0.05 indicates good model fit (Barrett, 2007). However, with large sample sizes chi-square typically over-rejects good fitting models (Tanaka, 1987). This occurs because chi-square is directly proportional to sample size, independent of the strength of the relationship between the variables. Accordingly, it is advisable to consider chi-square alongside other absolute fit indices (Hooper et al., 2008). In this context, SRMR and RMSEA values of 0.05 and lower indicate good fit; values between 0.06–0.08 signify acceptable fit; and 0.08–0.10 marginal fit (Browne and Cudeck, 1993). To assist interpretation of RMSEA, this study employed the 90 confidence interval (CI). CFI was included as a measure of incremental (or relative) fit. These indices compare the observed chi-square value to a baseline model. In this context, CFI is less sensitive to sample size. CFI assumes that all latent variables are uncorrelated (null model) and compares the sample covariance matrix with this null model. CFI values above 0.86 imply marginal fit (Bong et al., 2013), above 0.90 acceptable, and greater than 0.95 good fit (Hu and Bentler, 1999).

### Scale Properties and Inter-measure Correlations

Examination of inter-variable zero-order correlations revealed that RT correlated significantly with UL and belief in the paranormal (RPBS, NAP, and TPB) (see **Table 1**). Belief in the paranormal and UL were most strongly associated with the cognitive-perceptual and disorganized factors of schizotypy. Subsequently, CFA and SEM analyses focused on only the cognitive-perceptual and disorganized factors.

**Table 1:** Scale descriptive statistics and correlations.

	Mean	*SD*	1	2	3	4	5	6	7	8	9
(l) C-P	2.65	2.05		0.42ˆ**	0.44ˆ**	0.80ˆ**	0.65ˆ**	0.48ˆ**	0.44ˆ**	0.43ˆ**	0.27ˆ**
(2) lot	2.90	2.40			0.31ˆ**	0.80ˆ*	0.33ˆ**	0.10	0.07	0.16ˆ*	0.11
(3) Dis	1.71	1.71				0.70ˆ**	0.58ˆ**	0.18ˆ**	0.14ˆ*	0.19ˆ**	0.19ˆ**
(4) SPQ	7.27	4.76					0.67ˆ**	0.33ˆ**	0.28ˆ**	0.34ˆ**	0.24ˆ**
(5) RT	32.77	9.69						0.41ˆ**	0.37ˆ**	0.38ˆ**	0.34ˆ**
(6) RPBS	44.47	3044							0.89ˆ**	0.87ˆ**	0.28ˆ**
(7) NAP	19.76	6.41								0.76ˆ**	0.29ˆ**
(8) TPB	21.01	6.07									0.27ˆ**
(9) UL	20.38	7.20									

*C-P, Cognitive-Perceptual; Int, Interpersonal; Dis, Disorganized; SPQ-T, Schizotypy Personality Questionnaire-Total; RT, Reality Testing; RPBS, Revised Paranormal Belief Scale; NAP, New Age Philosophy; TPB, Traditional Paranormal Belief; UL, Urban Legends; ^∗^*p* < 0.05, ^∗∗^*p* < 0.001.*

### Confirmatory Factor Analysis

Prior to testing structural models, measurement scales were evaluated using research-informed solutions. The UL (Dagnall et al., 2010) and RT (Lenzenweger et al., 2001) scales are unidimensional. The RPBS comprises a correlated two-factor solution (TPB and NAP) (Lange et al., 2000). The SPQ-B contains three factors: cognitive-perceptual, disorganized and interpersonal (Raine and Benishay, 1995), although analysis excluded the interpersonal factor because it correlated (weakly) with only one belief measure (TPB).

The UL scale reported acceptable data-model fit, χ^2^(14, *N* = 222) = 20.47, *p* = 0.11, CFI = 0.98, SRMR = 0.03, RMSEA = 0.04 (CI of 0.01 to 0.08). The correlated RPBS two-factor model possessed unacceptable fit on all indices but SRMR, which was acceptable, χ^2^(103, *N* = 222) = 697.84, *p* < 0.001, CFI = 0.77, SRMR = 0.08, RMSEA = 0.16 (CI of 0.15 to 0.17). This finding was consistent with recent research demonstrating that the two-factor RPBS model yields marginal or unacceptable fit (Dagnall et al., 2016a). Data-model fit improved by allowing specific within-factor errors to correlate (items 8 and 22, 2, 9, and 16, and 7 and 14), χ^2^(98, *N* = 222) = 348.19, *p* < 0.001, CFI = 0.90, SRMR = 0.05, RMSEA = 0.10 (CI of 0.09 to 0.12).

Byrne (2013) contends that correlating within-item errors is permissible if a clear rationale exists. In the current study, the approach was valid because item combinations were consistent with subscales belonging to the original seven-factor RPBS solution (Tobacyk, 2004), specifically traditional religious belief (items 8 and 22), precognition (items 7 and 14) and psi (items 2, 9, and 16). Despite risking capitalization on chance (MacCallum et al., 1992), this approach aided interpretation. Capitalization on chance occurs when the characteristics of a sample influence the modifications performed and consequently, fail to generalize to other samples or the population. The correlated two-factor solution for schizotypy indicated acceptable fit on all indices but CFI, which indicated marginal fit, χ^2^(74, *N* = 222) = 133.82, *p* < 0.001, CFI = 0.89, SRMR = 0.06, RMSEA = 0.06 (CI of 0.04 to 0.07).

The unidimensional solution for RT reported unacceptable fit for CFI, whilst for SRMR and RMSEA fit was acceptable and marginal respectively, χ^2^(169, *N* = 222) = 512.67, *p* < 0.001, CFI = 0.76, SRMR = 0.07, RMSEA = 0.10 (CI of 0.09 to 0.11). This finding was consistent with the lack of consensus pertaining to the underlying structure of RT. Lenzenweger et al. (2001) proposed a unidimensional structure but found an appropriate two-factor solution. In addition, Ellison and Levy (2012) reported poor understanding of IPO psychometric properties and noted that a satisfactory solution currently does not exist.

Accordingly, performance of exploratory factor analysis (EFA) with oblique (promax) rotation) recommended an appropriate model. Byrne (2013) advocates EFA when no strong conceptual underpinning is present. EFA produced a four-factor solution (all loadings > 0.4) accounting for 55% of variance. Factor 1 (items 2, 5, 7, 8, 9, and 16) comprised statements related to ‘auditory and visual hallucinations’; factor 2 (items 11, 12, 14, 15, 17, 18 and 19) encompassed ‘delusional thinking’ (possessing beliefs contrary to reality); factor 3 (items 4, 10, 13, and 20) assessed ‘social deficits’ (difficulties reading social cues) and factor 4 (items 1, 3, and 6) corresponded to sensory/perceptual ‘confusion’ (inability to understand feelings and sensations). Identification of factors was consistent with the theoretical underpinnings of RT deficits (Bell et al., 1985; Caligor and Clarkin, 2010). Factors rotated obliquely in EFA and strong inter-factor correlations existed (between 0.42 and 0.49), henceforth a high-order four-factor solution was modeled (Gorsuch, 1983). CFA revealed acceptable data-model fit, χ^2^(165, *N* = 222) = 308.40, *p* < 0.001, CFI = 0.90, SRMR = 0.06, RMSEA = 0.06 (CI of 0.05 to 0.07). Subsequently, structural analyses used this model.

Overall, UL and schizotypy possessed factor solutions congruent with supporting theory. The RPBS explanation was consistent with the theoretical underpinning proposed by Lange et al. (2000), but required within-item error correlations due to the influence of original subscales. EFA and CFA assessments of the RT subscale supported a four-factor solution. The appropriateness of these factorial solutions can be further determined by examining parameter estimates. Factor loadings were positive and statistically significant. The majority of items possessed factor loadings greater than 0.60, meeting the strict requirements of Hair et al. (1998); all were above the minimum threshold of 0.32 (Tabachnick and Fidell, 2001).

### Composite Reliability

For latent modeling, traditional measures of internal reliability (e.g., Cronbach’s α) do not typically provide accurate indicators of scale reliability (Raykov, 2002). For this reason, composite reliability is preferred. Composite reliability provides a more rigorous measure of internal consistency, with values above 0.60 regarded as satisfactory (Diamantopoulos and Siguaw, 2000). In the current study, UL, NAP, and TPB demonstrated satisfactory composite reliability (ρ*c* = 0.69, ρ*c* = 0.73, and ρ*c* = 0.91, respectively). Cognitive-perceptual reported composite reliability below 0.60, however, the value was close to acceptable (ρ*c* = 0.58). Disorganized reported satisfactory composite reliability (ρ*c* = 0.65), as did all four sub-factors of RT (ρ*c* = 0.73, ρ*c* = 0.66, ρ*c* = 0.65, and ρ*c* = 0.67, respectively).

### Model Test: Schizotypy, Urban Legends, and Paranormal Beliefs

**FIGURE 1:**
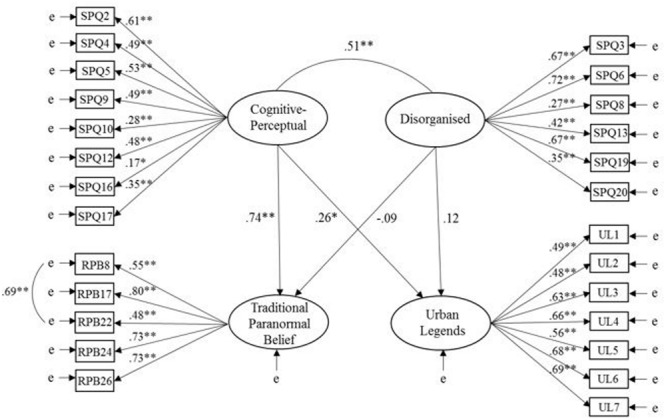
Model 1: relationships of cognitive-perceptual and disorganized schizotypy factors with traditional paranormal belief and urban legends (ULs). Latent variables are represented by ellipses; observed variables are represented by rectangles; error of measurement is indicated by ‘e’; ^∗^*p* < 0.05, ^∗∗^*p* < 0.001.

Model 1 (see **Figure 1**) produced acceptable to good data-model fit on all indices but CFI, which indicated marginal fit, χ^2^(289, *N* = 222) = 461.33, *p* < 0.001, CFI = 0.89, SRMR = 0.07, RMSEA = 0.05 (CI of 0.04 to 0.06). A comparison of structural paths between cognitive-perceptual factors and disorganized schizotypy factors with UL and TPB indicated that cognitive-perceptual had a significant positive effect on both TPB (β = 0.74, *p* < 0.001) and UL (β = 0.26, *p* = 0.02). Disorganized did not have a significant effect on TPB (β = -0.09, *p* = 0.37) or UL (β = 0.12, *p* = 0.26). The model accounted for 16% of the variance in TPB and 48% of the variance in UL. Disorganized correlated positively with cognitive-perceptual (*r* = 0.51, *p* < 0.001).

Model 2 demonstrated acceptable fit for CFI and SRMR, and good fit for RMSEA, χ^2^(451, *N* = 222) = 687.46, *p* < 0.001, CFI = 0.92, SRMR = 0.07, RMSEA = 0.05 (CI of 0.04 to 0.06). Similar to Model 1, a comparison of structural paths between cognitive-perceptual factors and disorganized with UL indicated that cognitive-perceptual had a significant positive effect on UL (β = 0.27, *p* = 0.01). Disorganized demonstrated a non-significant effect on UL (β = 0.21, *p* = 0.23). In contrast with Model 1, disorganized had a significant negative effect on NAP (β = -0.12, *p* = 0.03) and cognitive-perceptual had a significant positive effect on NAP (β = 0.84, *p* < 0.001). It is unclear why disorganized demonstrated a significant negative effect, but it is likely due to a confounding effect. Disorganized demonstrated a positive relationship with NAP once the cognitive-perceptual path was fixed to zero. Overall, the analysis supported the superior effect of cognitive-perceptual on NAP. Model 2 accounted for 12% of the variance in UL and 58% of the variance in NAP. Disorganized correlated positive correlation with cognitive-perceptual (*r* = 0.48, *p* < 0.001).

### Model Test: Reality Testing, Urban Legends, and Paranormal Beliefs

**FIGURE 2:**
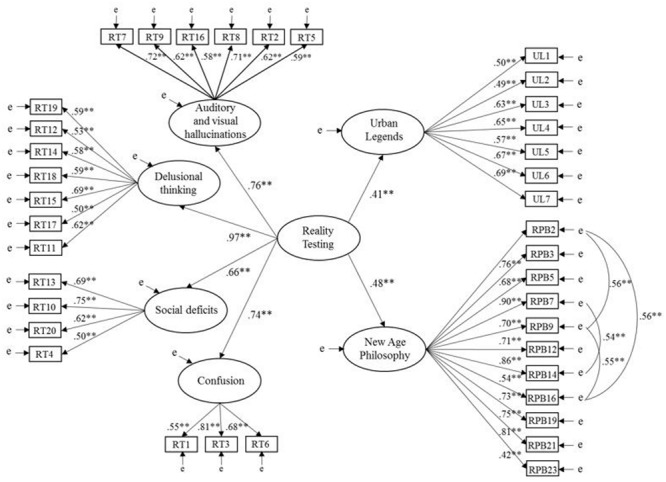
Model 4: relationships of reality testing with new age philosophy and urban legends. Latent variables are represented by ellipses; observed variables are represented by rectangles; error of measurement is indicated by ‘e’; ^∗^*p* < 0.05, ^∗∗^*p* < 0.001.

Model 3 (see **Figure 2**) demonstrated acceptable data-model fit for CFI and SRMR, and good fit for RMSEA, χ^2^(457, *N* = 222) = 706.29, *p* < 0.001, CFI = 0.90, SRMR = 0.07, RMSEA = 0.05 (CI of 0.04 to 0.06). RT had a significant positive effect on both UL (β = 0.41, *p* < 0.001) and TPB (β = 0.54, *p* < 0.001). Model 3 explained 29% of variance in TPB and 17% of variance in UL. Model 4 reported acceptable fit for CFI and SRMR, and good fit for RMSEA, χ^2^(655, *N* = 222) = 1016.17, *p* < 0.001, CFI = 0.90, SRMR = 0.07, RMSEA = 0.05 (CI of 0.04 to 0.06). Similar to Model 3, RT had a significant positive effect on both UL (β = 0.41, *p* < 0.001) and NAP (β = 0.48, *p* < 0.001). Model 4 accounted for 23% of variance in NAP and 17% of variance in UL.

## Conclusion

Findings support the notion that RT and the cognitive-perceptual factor of schizotypy strongly predict endorsement of UL and belief in the paranormal (TPB and NAP). Model comparison based on respective fit indices and predictive power indicated that schizotypy (Model 1 and 2) and RT models (Model 3 and 4) fitted well.

Reality testing explained a marginally greater amount of variance in UL, whilst schizotypy explained more variance in paranormal beliefs. Partial correlation supported this conclusion. Cognitive-perceptual and RT related differentially to endorsement of ULs and belief in the paranormal. Considering, ULs first. The relationship between RT and UL remained significant after controlling for cognitive-perceptual, *r* = 0.23, df = 219, *p* < 0.001. Contrastingly, the correlation between cognitive-perceptual and UL, controlling for RT, was non-significant, *r* = 0.06, df = 219, *p* < 0.001. In the case of belief in the paranormal, controlling for measure contribution reduced correlation size but relationships remained significant. The association between cognitive-perceptual and RPS, controlling for RT, was significant, *r* = 0.31, df = 219, *p* < 0.001. Similarly, the correlation between RT and RPBS, controlling for cognitive-perceptual, was weaker but still statistically significant, *r* = 0.15, df = 219, *p* = 0.013.

## Discussion

Proneness to RT deficits and the cognitive-perceptual factor of schizotypy strongly predicted endorsement of anomalistic beliefs (ULs and belief in the paranormal). This finding supported the view that cognitive-perceptual characteristics connected to positive schizotypy (i.e., magical ideation, odd beliefs, unusual experiences, and referential thinking) incline individuals toward validation of unusual beliefs. Generally, results were consistent with previous research reporting positive associations between proneness to RT deficits and corroboration of unconventional beliefs (ULs and the paranormal) (Drinkwater et al., 2012), and studies delineating relationships between positive schizotypy and belief in the paranormal (Genovese, 2005; Hergovich et al., 2008; Dagnall et al., 2010; Dembińska-Krajewska and Rybakowski, 2014). Contrastingly, the disorganized and interpersonal aspects of schizotypy demonstrated only weak and negligible (mainly non-significant) relationships respectively. These outcomes were congruent with the supposition that disorganized and interpersonal factors do not contribute directly to the formation of paranormal beliefs (Hergovich et al., 2008; Dagnall et al., 2010).

With regard to global cognitive style, there was a strong positive correlation between RT and the cognitive-perceptual factor; the measures shared approximately 42% variance. This relationship represented conceptual overlap and marked intuitive-experiential thinking style as an attendant feature of positive schizotypy. Inspection of cognitive-perceptual characteristics and previous investigations support this notion. Specifically, studies report a link between referential thinking, the tendency to find self-relevant meaning within random events, and belief in the paranormal (King and Hicks, 2009). This corresponds with Williams and Irwin’s (1991) proposition that belief in the paranormal arises from an individual’s attempts to structure the world in terms of person-centered, magical causality.

The idea that subjective/personal experiences guide and structure interpretation of information concurs also with CEST (Epstein, 1990, 1994). However, there are important issues to consider when applying and interpreting findings based on dual-processing models. Dual accounts of cognitive functioning and personality provide a conceptual framework for explaining individual differences in information processing style. The existence of two parallel, but interacting modes of cognition (analytical-rational and experiential-intuitive) offers a cogent framework in which to interpret reasoning inconsistencies. Emergent finding though, must acknowledge the existence of varying features and properties within individual dual-processing models.

Cognitive-Experiential Self-Theory differs from other dual-process models in important ways (Lu, 2015), which make it especially applicable to research investigating relationships between thinking and irrational beliefs/behaviors (Denes-Raj and Epstein, 1994). Particularly, because CEST evolved from the application of the dual-processing paradigm to personality it focuses on ‘thinking style.’ This approach contrasts with cognitive models, which view processing differences in terms of separate cognitive constructs (Pacini and Epstein, 1999). Within CEST, cognitive style denotes a preference for rational or intuitive processing. More generally, ‘thinking style’ refers to the use of heuristics to direct information processing (Kozhevnikov, 2007).

Cognitive-Experiential Self-Theory shares important parallels with cognitive, dual-processing frameworks that differentiate between controlled and automatic processing. These delineate the rational system as slow, effortful and demanding of attention, whilst the intuitive system is automatic, fast and non-conscious (Epstein, 2003). Despite apparent similarities, the terminology employed is not directly comparable. In particular, CEST is characterized by features that go beyond those typically considered by equivalent dual-process cognitive models, or frameworks. For instance, the notion that the experiential system is emotional and holistic is specific to CEST (Epstein, 1994). In addition, unlike dual-process cognitive accounts, CEST includes an unconscious system analogous to that outlined in psychoanalysis (Epstein, 2003).

With reference to CEST and the findings of the present study, cognitive-perceptual measures were highly correlated. This reflected the fact that they assessed a broader range of constructs than those typically captured by the traditional controlled and automatic processing distinction. Consequently, inter measure correlations may represent also variations within the dimensions of rational-experiential thought. This supposition is consistent with previous work, which denotes difficulties associated with interpreting and comparing dual-process model findings. Particularly, definitions between models vary and often lack precision. Hence, the general dual-processing literature lacks coherence and consistency (Evans and Stanovich, 2013). Accordingly, critics argue that findings are frequently explicable via alternative single-process models (Keren and Schul, 2009; Kruglanski and Gigerenzer, 2011). For example, Hayes et al. (2003) argue that there is no empirical support for the existence of two independent cognitive styles within CEST. They contend that research supports the view that there is a single bipolar intuition-analysis information processing system directed by a common set of principles driven by people’s daily cognitions (Hayes et al., 2003). Despite these concerns, considerable converging evidence from a range of psychological sub-disciplines supports the legitimacy of the dual-processing distinction (Evans and Stanovich, 2013).

Within the present study, it appears that believers are more likely to engage in experiential processing and base inferences about the world on intuition and self-generated perceptions. Thinking style undermines critical rational processing, which in turn perpetuates self-validation of beliefs. Thus, CEST applied to the study of the paranormal can explain why paranormal beliefs obstruct logical thinking (Wierzbicki, 1985) and despite limitations, provides a useful theoretical framework for understanding paranormal belief (Lasikiewicz, 2016).

Whilst endorsement of ULs and belief in the paranormal appear similarly related to proneness to RT deficits and the cognitive-perceptual factor, analysis revealed subtle differences. Particularly, validation of ULs was more strongly associated with RT, whilst the cognitive-perceptual factor best explained belief in the paranormal. This observation ran contrary to the notion that anomalous beliefs arise predominantly from defiance of conventional understanding of reality (French and Stone, 2013; Brotherton and French, 2014). Whilst, intuitive thinking style and proneness to RT deficits is a feature of unconventional beliefs, it is not a determining characteristic of anomalous beliefs generally. From a cognitive-perceptual perspective, endorsement of specific unusual beliefs is associated with a range of variables and the relative importance of these varies across belief types. Indeed, within the present study ULs and belief in the paranormal shared only approximately 8% variance. This figure was similar to that reported previously by Dagnall et al. (2010). In this context, it is appropriate to conclude that whilst the size of relationship between endorsement of ULs and belief in the paranormal varies as a function of measure, the correlation is at best moderate (Drinkwater et al., 2012). Additionally, the findings suggest different combinations of cognitive-perceptual best explain individual anomalistic beliefs. Therefore, anomalous beliefs are best approached and understood on a phenomenon-by-phenomenon basis.

In the case of ULs, proneness to RT deficits may play a prominent role because authentication derives largely from uncritical acceptance of unsubstantiated information; essentially, the reader accepts prima facie evidence as accurate/truthful. Uncritical acceptance based on emotional rather than rational appeal is a key feature of intuitive-experiential thinking (Epstein, 1990, 1994). In this sense, the recipient of an UL makes a judgment about the supposition of alleged causal relationships based on presented information. Individuals adopting an analytical-rational processing style are predisposed to critically consider information (evidence, experiences, thoughts, etc.) and accordingly more likely to reject ULs (Dagnall et al., 2010).

Ratification of general paranormal beliefs differs because it stems principally from inferring and elucidating connections between events, occurrences and happenings. The key factor is generation, justification, and explanation of causal relationships between random (non-related) factors. Although, it is important to note that there is empirical evidence in support of paranormal phenomena (i.e., precognition and premonition, Bem, 2011). The observation that proneness to reality deficits was a stronger predictor of endorsement of ULs than belief in the paranormal was consistent with Dagnall et al. (2010).

The cognitive-perceptual factor may best explain variance within belief in the paranormal because it is a broader construct. Paranormal beliefs are more diverse and less homogeneous than ULs. Specifically, although individual ULs vary in plausibility, acceptance arises from the same cognitive processes (uncritical acceptance of unsubstantiated information as accurate) (Dagnall et al., 2010). In the case of paranormal beliefs, phenomena vary in both credibility and underlying causation. For example, psi relies on inferred causation, whilst the existence of some extraordinary life forms (i.e., ‘Loch Ness Monster’ and ‘Abominable Snowman of Tibet’) arises more from the internalization of social myths and pseudo-scientific thought. In this context, the characteristics subsumed with the cognitive-perceptual factor provide a better explanation of belief in paranormal phenomena generally compared to RT. The tendency to focus on general belief, rather than specific beliefs, reflects factorial inadequacies within the two main measures (R-PBS and ASGS) and researchers’ general preference to assess overall belief. Consideration of specific beliefs would allow subsequent work to test this hypothesis.

Concerning RT, this study provided qualified support for Irwin’s (2003, 2004) supposition that anomalous beliefs are associated with an intuitive-experiential thinking (processing style) and the failure to appraise evidence, experiences and thoughts to critical analytical-rational processing. It appears that the importance of thinking style varies as a function of belief type. Hence, assumptions based on perceived surface level similarities are limited. For these reasons, further research needs to assess the degree to which this is true for particular specific beliefs. Comparable research conducted with schizotypy concluded that the constructs relationship with paranormal beliefs and experiences was complex (Irwin and Green, 1998-1999).

Another useful extension may be to compare endorsement of unusual, but real and reported events with traditional and manufactured ULs. For example, it would be interesting to see whether endorsement rates vary across event types and if proneness to RT deficits predict response rates. Clearly, respondents scoring highly should be more likely to endorse accounts regardless of nature and/or underlying veracity. Topically, this research could also extend to the consideration of fake news (widespread disseminate of inaccurate, false and/or created news).

An important limitation of the present study concerned the manner in which the IPO-RT assessed RT. Principally, the scale indexed the subjective evaluation of perceived likelihood of RT errors. Accordingly, measurement validity relied on the extent to which IPO-RT items enabled individuals to distinguish between potential sources of information (internal vs. external) (Garrison et al., 2017). This approach contrasted with experimental-based performance measures, which ‘actually’ assess the accuracy of RT; tasks demand that participants discriminate between perceived and imaged events (Johnson and Raye, 1981, 1998; Johnson, 1988, 1997).

The distinction between external and internal generated events is inherent within the broader concept of source monitoring (Johnson et al., 1993). Specifically, the source-monitoring framework (SMF) (Johnson et al., 1993; Mitchell and Johnson, 2009). The SMF postulates that the origins of thoughts, sensations and memories are not tagged as belonging to one (vs. another) source. Instead, labels, such as internal or external, result from an attributional process. Attributions arise from assumptions about the typical features that characterize a source. Thus, external designations are more likely if visual, or other sensory details are associated with recalled material. Unfortunately, features that generally constitute evidence for a source are neither absolute, nor infallible. Misattributions arise as a function of strategic and heuristic/automatic processing and occur often when retrieved contents possess features that share commonality with an alternative source (Marsh et al., 1997). Due to the general and spontaneous nature of RT decisions, individuals may lack conscious awareness, or insight into judgment processes and their veracity. This is likely to affect self-report measure accuracy.

Future work could assess objectively the ability of participants to make accurate reality monitoring judgments, both in relation to other established self-report measures and factors known to produce errors. The inclusion of experimental-based performance measures is important because they provide an objective measure against which to assess IPO-RT scores. This is required because previous research has demonstrated that self-report measures of a cognitive process do not always correspond with experimental assessments and/or performance. For instance, self-report measures of metamemory often fail to predict mnemonic performance (Sunderland et al., 1986). If subjective and objective measures of RT do not align, it will be important to evaluate why this is the case. This is especially true if observed differences relate also to other subjective measures. Conceptually, it is important to ensure thorough understanding of the nature and limitations of RT procedures and measures. Once established researchers will be able to determine precisely the degree to which factors influencing reality monitoring interact with specific anomalous beliefs.

Another potential limitation within the present study was the fact that measures demonstrated an element of conceptual overlap. Particularly, the cognitive-perceptual factor of the SPQ-B contained items related to odd beliefs or magical thinking and unusual perceptual experiences, these indexed both belief in the paranormal and IPO-RT content. For example, two items evaluating odd beliefs or magical thinking related to paranormal beliefs. In one instance the correspondence was direct (“Have you had experiences with astrology seeing the future. UFOs, ESP, or a sixth sense?”), whilst in the other the link was indirect and inferential (“Are you sometimes sure that other people can tell what you are thinking?”). In the case of the second item, endorsement would only indicate belief in the paranormal (presumably extrasensory perception) if the internal explanation/attribution provided for the perceived phenomenon referenced the paranormal.

Examination of unusual perceptual experiences items revealed potential intersection with IPO-RT content (e.g., “Do you ever suddenly feel distracted by distant sounds that you are not normally aware of?”). The second unusual perceptual experiences question potentially indexed both belief in the paranormal and proneness to RT deficits (“Have you ever had the sense that some person or force is around you even though you cannot see anyone?”). Conceptual overlap is inevitable when related constructs are included within test batteries. In the present study, whilst associated, the two cognitive-perceptual measures served different functions. The SPQ-B acted as a dispositional measure of cognitive-perceptual preference, whilst the IPO-RT indexed preferential thinking style. Additionally, theoretically and psychometric both schizotypy and proneness to RT deficits have been validated as separate, independent constructs.

Overall, the lack of shared variance between belief measures together with their differential interactions with cognitive-perceptible factors provides compelling evidence to reject the notion that anomalous beliefs are locatable within a generic category. Future studies may wish to consider construct overlap when designing studies. This is achievable via psychometric evaluation of individual items, and/or omission of items where clear theoretical intersection is evident, providing of course that this does not damage psychometric integrity.

Findings from the present study were important for myriad reasons. Firstly, they contribute to the expanding literature examining relationships between cognitive-perceptual factors and anomalous beliefs. Secondly, results provided further insights into the contribution of intuitive thinking style and schizotypy to the formation and maintenance of anomalous beliefs. Finally, the paper explored the relationship between proneness to RT and schizotypy. This was important because the RTS and SPQ-B, explain difference nuances within anomalous beliefs.

## Ethics Statement

The researcher obtained ethical approval for the study as part of a research proposal examining the relationship between anomalous beliefs and cognitive-perceptual measures. This was approved by the Director of the Research Institute for Health and Social Change and supported by the Departmental Head. In order to be submitted all bids are first required to obtain ethical clearance. This involves scrutiny via peer-review and the Director of the Research Institute for Health and Social Change. Reviewers have in-depth understanding of ethical requirements/considerations are typically members of the University Professoriate.

## Author Contributions

KD and ND designed the study, organized data collection, supported analysis and were main authors. AD conducted main analysis and was a contributing author. AP contributed to the writing process. PC advised on paper and assisted with drafting.

## Conflict of Interest Statement

The authors declare that the research was conducted in the absence of any commercial or financial relationships that could be construed as a potential conflict of interest.

## Footnotes

1 http://www.snopes.com
